# Demographic, Clinical, and Laboratory Characteristics of Pediatric IgA Vasculitis: A Retrospective Five-Year Single-Center Experience

**DOI:** 10.3390/jcm15041508

**Published:** 2026-02-14

**Authors:** Mukaddes Kılıç Sağlam, Müferet Ergüven

**Affiliations:** 1 Department of Pediatrics, Faculty of Medicine, Duzce University, 81620 Duzce, Türkiye; 2 Department of Pediatric Rheumatology, Faculty of Medicine, Duzce University, 81620 Duzce, Türkiye; muferete@yahoo.com

**Keywords:** Henoch–Schönlein purpura, Immunoglobulin A vasculitis, purpura, child, vasculitis

## Abstract

**Background**: Immunoglobulin A (IgA) vasculitis is the most common vasculitis of childhood. This study aimed to evaluate the demographic characteristics, clinical manifestations, laboratory findings, prognostic features, differential diagnoses, and treatment approaches of children diagnosed with IgA vasculitis in our clinic. **Methods**: This retrospective study included children diagnosed with IgA vasculitis between February 2020 and November 2025. Demographic, clinical, laboratory, imaging, biopsy, and treatment data were obtained from medical records. **Results**: Seventy-four patients were included (mean age: 7.7 ± 3.3 years; 60.8% female), with admissions occurring most frequently in autumn and winter. A preceding infection within the last two weeks was present in 78% of patients. *Epstein–Barr virus* IgM positivity was detected in three patients and *Cytomegalovirus* IgM positivity in three patients. Joint, gastrointestinal, and renal involvement were observed in 35, 46, and 30 patients, respectively; testicular involvement was detected in two patients and pneumonic infiltration in one patient. Severe gastrointestinal involvement was observed in six patients (melena in four and intussusception in two). Extensive rheumatologic testing revealed no additional pathology, and skin punch biopsy demonstrated findings consistent with IgA vasculitis in all cases. Corticosteroids were required in 44 patients due to gastrointestinal or renal involvement, or persistent disease. **Conclusions**: Although IgA vasculitis is generally self-limiting, careful clinical monitoring is essential due to the risk of acute gastrointestinal, testicular, and renal complications, and its potential to mimic other causes of acute abdomen before purpura onset. Extensive rheumatologic testing, broad infectious screening and skin biopsy did not provide additional diagnostic or follow-up value beyond clinical assessment.

## 1. Introduction

Immunoglobulin A vasculitis (IgAV), formerly known as Henoch–Schönlein purpura, is the most common vasculitis of childhood. The disease is characterized by a systemic small-vessel vasculitis with predominant IgA1-containing immune-complex deposition within affected small vessels, including the renal glomeruli [1]. Although it is seen predominantly in children, it may also occur in adults. The clinical presentation typically involves cutaneous, gastrointestinal (GIS), articular, and renal manifestations. Patients may present with non-thrombocytopenic palpable purpura, GIS bleeding, abdominal pain, vomiting, arthralgia, arthritis, and nephritis. In most cases, the disease follows a self-limiting course and resolves within approximately four weeks; however, less common organ involvements such as testicular, pulmonary, cardiac, or neurological complications may also occur [2].

Short-term prognosis is largely determined by the severity of gastrointestinal involvement, whereas long-term outcomes depend primarily on the extent of renal involvement. The annual incidence of IgA vasculitis in children has been reported as 20 per 100,000 [3]. Although its exact etiology has not been fully elucidated, genetic predisposition, environmental factors, and infectious agents are thought to contribute to disease development. Various triggers, including infectious pathogens, medications, foods, and vaccines, have been implicated in the onset of IgA vasculitis [4].

In this study, we aimed to evaluate the demographic characteristics, clinical features, laboratory findings, and treatment approaches of children diagnosed with IgA vasculitis in our pediatric rheumatology clinic. Although numerous studies on IgA vasculitis are available in the literature, extensive diagnostic investigations—including broad rheumatologic testing, evaluation for streptococcal infection and other viral infectious triggers, as well as routine skin biopsy—are not commonly performed in the diagnostic workup of IgA vasculitis. By systematically including these assessments in our clinical practice, we aimed to contribute additional data to the literature and to better clarify their diagnostic and clinical utility in pediatric IgA vasculitis.

## 2. Materials and Methods

In this retrospective study, the demographic, clinical, laboratory, and treatment data of pediatric patients diagnosed with IgA vasculitis and followed in the Pediatric Rheumatology Clinic of Düzce University Faculty of Medicine between February 2020 and November 2025 were reviewed from medical records. Prior to the initiation of the study, ethical approval was obtained from the Düzce University Ethics Committee (Approval number: 2025/308; Date: 24 November 2025).

For each patient, age, sex, date of diagnosis, season of presentation, triggering factors, length of hospital stay, clinical characteristics (renal, gastrointestinal, joint, and other organ involvement, as well as the distribution of skin lesions), laboratory parameters and imaging findings at diagnosis (abdominal ultrasonography, chest radiography), and administered treatments were recorded. In our clinic, extensive rheumatologic investigations and skin punch biopsy are routinely performed in patients diagnosed with IgA vasculitis as part of the standard diagnostic workup to exclude alternative diagnoses. Therefore, these data were available for analysis in this retrospective study. Patients with another form of vasculitis or rheumatologic disease, or those with missing key clinical data in their files, were excluded from the study.

The diagnosis of IgA vasculitis was established according to the Ankara criteria [5]. Patients presenting with abdominal pain, vomiting, or a positive fecal occult blood test were classified as having mild GIS involvement, whereas those with melena, hematochezia, or intussusception were categorized as having severe GIS involvement [6]. Renal involvement was defined as the presence of microscopic or macroscopic hematuria, proteinuria, renal impairment, or hypertension. Microscopic hematuria was defined as the presence of more than five red blood cells per high-power field on microscopic urinalysis. Proteinuria was defined as a spot urine protein-to-creatinine ratio greater than 0.2 in children older than two years, and greater than 0.5 in those younger than two years [7].

Potential triggering factors included infections occurring within the two weeks preceding disease onset, consumption of specific foods, recent vaccinations, insect bites, or a history of trauma. An erythrocyte sedimentation rate (ESR) exceeding 20 mm/hr was considered elevated; a C-reactive protein (CRP) level above 0.5 mg/dL was classified as high; and an antistreptolysin-O (ASO) titer above 200 IU/mL was considered elevated [8]. Thrombocytosis was defined as a platelet count greater than 450,000/mm^3^ [9]. Age-specific reference ranges were used to assess leukocytosis and anemia [10].

Glucocorticoid therapy was administered to patients with severe abdominal pain, renal involvement, or significant gastrointestinal disease. Oral prednisone was used at a dose of 1–2 mg/kg/day, with a maximum daily dose of 60 mg. In patients who were unable to tolerate oral medication, parenteral methylprednisolone was administered at an equivalent dose of 0.8–1.6 mg/kg/day (maximum daily dose of 64 mg). For patients with life-threatening gastrointestinal or renal involvement, pulse corticosteroid therapy was applied for three consecutive days, in accordance with institutional clinical practice.

### Statistical Analysis

For continuous variables, descriptive statistics were expressed as mean ± standard deviation, median, minimum, and maximum values, whereas categorical variables were presented as percentages.

Fisher’s exact test was used to compare disease severity between patients who received a diagnosis within fewer than 7 days and those diagnosed at or after 7 days.

All analyses were performed using IBM SPSS for Windows version 20.0 (SPSS Inc., Chicago, IL, USA), and a *p*-value < 0.05 was considered statistically significant.

## 3. Results

A total of 74 patients were included in the study. The mean age was 7.72 ± 3.3 years (range: 1.3–15.5 years), and 60.8% of the patients were female. Seasonal distribution showed that the majority of admissions occurred in autumn (35.1%) and winter (31.1%). The median time to diagnosis was 3 days, and 73% of the patients received a diagnosis within the first 7 days. There was no significant difference in disease severity between patients diagnosed within <7 days and those diagnosed ≥7 days after symptom onset (*p* = 0.379). A history of infection within the preceding two weeks was present in 78% of the patients, most commonly upper respiratory tract infections (71.6%), followed by acute gastroenteritis (4.0%) and urinary tract infections (2.7%). The mean duration of hospitalization was 6.04 ± 4.05 days.

All patients presented with palpable purpura. In 62 patients, the rash was localized exclusively to the lower extremities, while two patients had additional facial involvement and two had truncal involvement. Gastrointestinal involvement was present in 46 patients. Four cases exhibited severe rectal bleeding, and two developed intussusception; however, none required surgical intervention. Joint involvement was observed in 35 patients—most commonly affecting the ankles—while subcutaneous edema was noted in 21 patients, and testicular involvement in 2 patients. Renal involvement developed in 30 patients, four of whom met the criteria for severe renal disease. Hypertension was detected in four patients, proteinuria in twenty, and hematuria in thirteen. Two patients with persistent nephrotic-range proteinuria underwent renal biopsy.

Nonsteroidal anti-inflammatory drugs (NSAIDs) were initiated in patients with joint involvement and/or abdominal pain and were used in 50% of the cohort. Corticosteroid therapy was administered to 59.5% of the patients due to gastrointestinal or renal involvement. Three patients with severe disease—two with severe gastrointestinal involvement and one with severe renal involvement—received pulse corticosteroid therapy for three consecutive days, followed by parenteral methylprednisolone and subsequent transition to oral prednisone after discharge. In fourteen patients, parenteral methylprednisolone was administered because oral therapy was not initially tolerated; these patients were switched to oral prednisone once oral intake became feasible. In the remaining patients, oral prednisone therapy was initiated and, when clinically indicated, continued after discharge based on clinical and laboratory findings. All patients who achieved remission were discharged with scheduled outpatient follow-up. The demographic and clinical characteristics of the patients are presented in Table 1.

In the laboratory evaluation, anemia was detected in 29 patients (39.2%), leukocytosis in 3 patients (4.1%), thrombocytosis in 26 patients (35%), elevated ESR in 50 patients (67.6%), and elevated CRP levels in 44 patients (59.5%). An increase in serum creatinine was identified in one patient. Viral serology showed positivity in six patients (anti-Cytomegalovirus (CMV) IgM: *n* = 3; *Epstein–Barr virus* (EBV) VCA IgM: *n* = 3). Anti-Toxoplasma IgM, Anti-Rubella IgM, Anti-HAV IgM, and HBsAg were negative in all patients.

Rheumatologic tests performed for differential diagnosis revealed negative Antinuclear Antibody (ANA) in 52 patients. Among cases in which borderline or equivocal ANA patterns were observed, one had a homogeneous pattern, one displayed an intercellular bridge-like staining pattern, 15 showed a fine speckled pattern, four had a coarse speckled pattern, and one demonstrated mitotic body positivity. However, ANA titers were below 1:160 in these patients, and systemic lupus erythematosus or other connective tissue diseases were excluded based on clinical assessment. Anti-double-stranded DNA (Anti-dsDNA) results (*n* = 61) and Anti-Neutrophil Cytoplasmic Antibody (ANCA) results (*n* = 47) were negative in all patients. Complement levels (C3 and C4), assessed in 64 patients, remained within normal limits.

Chest X-ray imaging demonstrated pneumonic infiltration in only one patient, while all others were normal. All Tuberculin skin tests were negative. Brucella serology, performed in 45 patients, was negative. Throat cultures were obtained from 26 patients, and none showed β-hemolytic streptococcal growth. Elevated ASO titers were detected in 22 patients (41.5%).

Skin punch biopsy was performed in 69 patients (93%). All biopsy samples showed findings consistent with IgA vasculitis, including perivascular inflammatory infiltration rich in polymorphonuclear leukocytes, mild fibrin deposition, karyorrhexis, and leukocytoclastic vasculitis. The laboratory findings of the patients are summarized in Table 2.

## 4. Discussion

IgA vasculitis is the most common vasculitis of childhood. Approximately 90% of IgA vasculitis cases occur in children aged 2–10 years, with a peak incidence between 4 and 7 years [11]. This age range corresponds to the period when children begin kindergarten and are exposed to numerous viral and bacterial infectious agents. In a large cohort study from Türkiye including 709 children, the mean age was reported as 7.9 ± 3.2 years, and 55% of the patients were male [12]. Another study found a mean age of 7.5 ± 3.2 years, with 51.7% of patients being male [13]. In our study, while the mean age was consistent with previous Turkish cohorts, we observed a higher proportion of female patients compared to the existing literature.

Although the etiology of IgA vasculitis has not been fully elucidated, various infectious and non-infectious triggers, including bacterial, viral, and parasitic infections; vaccinations; trauma; burns; insect bites; and certain medications and antibiotics, have been implicated in its pathogenesis [4,14]. Among the pathogens associated with IgA vasculitis, group A β-hemolytic streptococcus is the most extensively studied. Previous reports have shown that approximately 60% of IgA vasculitis cases may be associated with pneumococcal or group A streptococcal infections [15]. Furthermore, positive throat cultures have been reported in 10–30% of cases, whereas elevated ASO titers have been observed in 7.5–48.2% of patients [16]. Another study identified Mycoplasma pneumoniae as the most common infectious trigger, while the frequencies of group A streptococcus, EBV, and CMV were reported as 28.8%, 7%, and 6.3%, respectively [17]. The high frequency of upper respiratory tract infections preceding disease onset in our cohort (71.7%) is consistent with previous pediatric studies and further supports the hypothesis that respiratory infections play a key triggering role in IgA vasculitis. None of the throat cultures yielded growth. We speculate that this may be due to the inclusion of patients with recent infections who had received antibiotic therapy, resulting in culture sampling during the recovery phase. Consistent with the literature, elevated ASO titers were detected in 22 patients (31.5%). In our cohort, extensive viral and infectious screening was largely unremarkable, with only a small number of patients showing acute EBV or CMV seropositivity, while Brucella serology, HBsAg, and other viral markers were negative in all cases. These findings suggest that routine broad infectious screening may have limited diagnostic yield in typical pediatric IgA vasculitis and should be considered selectively based on clinical suspicion rather than performed systematically.

IgA vasculitis occurs most frequently during the autumn and winter seasons, while it is least common in the summer months [18]. The findings of our study are consistent with the existing literature. We believe that this seasonal pattern may be related to the seasonal distribution of infectious agents implicated in the disease etiology.

IgA vasculitis resolves spontaneously in approximately 94% of children and in most cases, the disease follows a self-limited course requiring only supportive management. However, a subset of patients may develop progressive renal involvement. Additionally, complications such as gastrointestinal bleeding, orchitis, and central nervous system involvement can occur [19]. The short-term prognosis largely depends on the severity of acute gastrointestinal involvement, whereas the long-term prognosis is determined by the extent of renal damage. Recent studies have shown that progression to end-stage renal disease may occur even more than 10 years after the initial onset of IgA vasculitis [2].

GIS involvement may present with abdominal pain, nausea, vomiting, melena, hematochezia, and intussusception. In a study from China, GIS involvement was reported in 50% of patients, with 24.4% demonstrating severe GIS manifestations [20]. In the study by Karadağ et al., the rate of GIS involvement was 51.3%, and severe involvement was observed in 12.9% of cases [13]. Occult blood positivity in stool has been reported at approximately 25.8% in previous studies [21]. Severe complications such as intussusception and bowel ischemia occur in about 1–5% of cases, depending on the study population and diagnostic criteria used [22]. In our study, the frequency of intussusception was consistent with the literature. Although the rates of GIS involvement and occult blood positivity were higher compared with previous reports, the prevalence of severe GIS involvement was relatively low at 5.5%. We believe this may be attributed to the fact that, despite being a tertiary care center, our hospital serves as the only pediatric rheumatology unit in the region, resulting in the referral or direct presentation of the majority of IgA vasculitis cases, including mild presentations, to our center.

Renal involvement in IgA vasculitis has been reported to range between 20% and 54% in various studies [23]. In our study, the renal involvement rate was 40.5%, which is consistent with published data.

Antinuclear antibodies (ANA) are generally reported to be negative in IgA vasculitis, and serum complement levels are typically within normal ranges, reflecting the immune complex-mediated rather than complement-consuming nature of the disease [24]. Nevertheless, transient hypocomplementemia has been described in a subset of pediatric patients. For example, Lin et al. reported decreased C3 and/or C4 levels in 15.7% of children, with spontaneous normalization within three months in nearly all cases [25]. In our cohort, ANA was negative in all patients, and complement levels remained within normal limits in all evaluated cases. This finding supports the concept that routine complement testing and extensive autoimmune serology may have limited diagnostic or prognostic value in typical presentations of pediatric IgA vasculitis. Moreover, the absence of persistent hypocomplementemia in our cohort suggests that complement abnormalities, when present, may represent a transient epiphenomenon rather than a marker of disease severity or chronicity. Our results therefore reinforce current evidence that clinical assessment remains central to diagnosis and follow-up, and that broad autoimmune testing should be reserved for selected cases with atypical features or alternative diagnostic considerations.

Most IgA vasculitis cases resolve spontaneously, and the majority of patients can be managed on an outpatient basis. Treatment is primarily supportive and includes adequate hydration, rest, and symptomatic analgesic therapy. Dependent edema—particularly in the lower extremities, hips, and genital region—often improves with bed rest and/or elevation of the affected area. Nonsteroidal anti-inflammatory drugs are commonly used for the symptomatic management of abdominal and joint pain.

There is no universal consensus regarding the indications for corticosteroid therapy in IgA vasculitis. Corticosteroids may be helpful in alleviating severe abdominal and joint pain and may be considered in the presence of bullous, ulcerative, or necrotic skin lesions. Corticosteroid therapy is generally considered in cases with severe gastrointestinal involvement or significant renal involvement. However, routine empirical glucocorticoid therapy is not routinely recommended for all patients with IgA vasculitis for the treatment of mild symptoms or for the prevention of renal or gastrointestinal complications. While glucocorticoid therapy has not been shown to have a clear preventive effect on renal involvement, some studies in the literature suggest potential benefits in reducing extra-renal complications. For example, early glucocorticoid treatment in children with IgA vasculitis has been reported to be associated with a reduced risk of gastrointestinal procedures, including endoscopy, imaging, and surgery [2,26].

In our study, corticosteroids were initiated in 44 patients (59.5%) due to severe abdominal pain, renal involvement, or significant gastrointestinal disease. No steroid-related complications were observed.

This study has several limitations. Due to its retrospective design, complete datasets were not available for all patients, and only cases with complete core data were included in the analysis. The study was limited to clinical findings at initial presentation and during hospitalization, as well as laboratory data obtained during this period. As data from the past five years were evaluated and long-term follow-up durations could not be obtained simultaneously for all patients, long-term outcomes such as relapse, recurrence, and late renal involvement could not be assessed. Furthermore, as this was a single-center study, the findings may have limited generalizability to broader populations due to potential regional and genetic variations.

## 5. Conclusions

Although IgA vasculitis follows a self-limiting course in most patients, close monitoring is essential due to the risk of serious gastrointestinal complications such as intussusception, as well as testicular involvement and renal impairment. In one of our cases, acute abdominal pain preceding the onset of purpura led to surgical intervention with a preliminary diagnosis of acute appendicitis. This observation highlights that IgA vasculitis can mimic other acute abdominal conditions, particularly in its early phase. It underscores the importance for clinicians to be aware of diagnostic challenges before purpura becomes apparent. Given the high likelihood of complications during the acute phase, thorough clinical evaluation is crucial. Our findings indicate that extensive rheumatologic panels and skin biopsy, which are not routinely recommended in HSP, do not contribute additional diagnostic or follow-up information. Skin biopsy findings merely supported the clinical diagnosis, and no additional abnormalities were identified in rheumatologic assessments. Routine broad infectious screening is not warranted in typical pediatric IgA vasculitis in the absence of clinical suspicion.

## Figures and Tables

**Table 1:** Demographic and Clinical Characteristics of the Patients.

*n* = 74	Mean ± SD Median (Min–Max)	
Age (year)	7.72 ± 3.31 7.2 (1.3–15.5)	
Time until diagnosis (day)	4.55 ± 4.61 3 (0–20)	
Day of steroid therapy initiation	6.83 ± 5.16 5 (1–21)	
Length of hospital stay (days)	6.04 ± 4.05 6 (0–19)	
	***n***	**%**
Gender		
Female	45	60.8
Male	29	39.2
Season of disease onset		
Spring	16	21.6
Summer	9	12.2
Autumn	26	35.1
Winter	23	31.1
Day of diagnosis		
<7 days	54	73.0
≥7 days	20	27.0
Steroid therapy		
Yes	44	59.5
No	30	40.5
Predisposing factor		
Upper respiratory tract infection	53	71.6
Gastroenteritis	3	4
Urinary tract infection	2	2.7
None	16	21.6
Purpura		
Lower extremities	62	83.8
Lower extremities + upper extremities	8	10.8
Lower extremities + upper extremities +Trunk	2	2.7
Lower extremities + upper extremities + Head	2	2.7
Joint involvement	35	47
Subcutaneous edema	21	28.3
Renal involvement None Mild Severe Proteinuria Hematuria High blood pressure	44 26 4 20 13 4	59.5 35 5.5 27 17.6 5.5
Gastrointestinal system (GIS) involvement		
None	28	37.8
Mild	42	56.8
Severe	4	5.5
Abdominal pain	46	62.2
Occult stool blood	40	54
Hematochezia/melena	4	5.5
İntussusception	2	2.7

**Table 2:** Laboratory findings of the patients.

	Mean ± SD Median (Min–Max) N (%)
Hemoglobin (g/dL) (*n* = 74)	11.74 ± 1.09 11.7 (9.1–14.4)
Anemia	29 (39.2%)
Platelet count (cells/µL) (*n* = 74)	414,554.05 ± 123,009.31 398,000 (203,000–802,000)
Thrombocytosis	26 (35%)
WBC count (cells/µL) (*n* = 74)	9898.38 ± 3012.33 9610 (4230–19,100)
Leukocytosis	3 (4.1%)
CRP (mg/dL) (*n* = 74)	1.45 ± 1.79 0.67 (0–7.7)
Elevated CRP	44 (59.5%)
ESR (mm/hr) (*n* = 74)	31.28 ± 19.16 30 (5–102)
Elevated ESR	50 (67.6%)
Creatinine (mg/dL) (*n* = 74)	0.44 ± 0.13 0.43 (0.08–0.88)
Elevated Creatinine	1 (1.4%)
C3 (mg/dL) (*n* = 64)	137.69 ± 25.76 137 (83–189)
C4 (mg/dL) (*n* = 64)	26.23 ± 6.79 25 (13–43)
ASO (IU/mm) (*n* = 53)	416.68 ± 845.83 160 (0.27–5412.0)
Elevated ASO level	22 (41.5%)
*Epstein–Barr virus* (EBV) VCA Ig M positivity (*n* = 67)	3 (4.5%)
Anti-*Cytomegalovirus* (CMV) Ig M positivity (*n* = 67)	3 (4.5%)

ESR: Erythrocyte sedimentation rate; CRP: C-reactive protein; ASO: Antistreptolysin-O; C3: Complement C3; C4: Complement C4.

## Data Availability

The raw data supporting the results of this study are available upon reasonable request from the corresponding author. Due to ethical restrictions, the data can only be shared in an anonymized form. To ensure compliance with institutional regulations, a data transfer agreement may be required prior to sharing the data.
